# Predicting the Rheological Properties of Super-Plasticized Concrete Using Modeling Techniques

**DOI:** 10.3390/ma15155208

**Published:** 2022-07-27

**Authors:** Muhammad Nasir Amin, Ayaz Ahmad, Kaffayatullah Khan, Waqas Ahmad, Saqib Ehsan, Anas Abdulalim Alabdullah

**Affiliations:** 1 Department of Civil and Environmental Engineering, College of Engineering, King Faisal University, Al-Ahsa 31982, Saudi Arabia; kkhan@kfu.edu.sa (K.K.); 218038024@student.kfu.edu.sa (A.A.A.); 2 MaREI Centre, Ryan Institute and School of Engineering, College of Science and Engineering, National University of Ireland Galway, H91 TK33 Galway, Ireland; a.ahmad8@nuigalway.ie; 3 Department of Civil Engineering, COMSATS University Islamabad, Abbottabad 22060, Pakistan; waqasahmad@cuiatd.edu.pk; 4 Department of Civil Engineering, NFC Institute of Engineering and Fertilizer Research, Faisalabad 38090, Pakistan; saqib.ehsan@iefr.edu.pk

**Keywords:** concrete, plastic viscosity, yield stress, modeling, machine learning

## Abstract

Interface yield stress (YS) and plastic viscosity (PV) have a significant impact on the pumpability of concrete mixes. This study is based on the application of predictive machine learning (PML) techniques to forecast the rheological properties of fresh concrete. The artificial neural network (NN) and random forest (R-F) PML approaches were introduced to anticipate the PV and YS of concrete. In comparison, the R-F model outperforms the NN model by giving the coefficient of determination (R^2^) values equal to 0.92 and 0.96 for PV and YS, respectively. In contrast, the model’s legitimacy was also verified by applying statistical checks and a k-fold cross validation approach. The mean absolute error, mean square error, and root mean square error values for R-F models by investigating the YS were noted as 30.36 Pa, 1141.76 Pa, and 33.79 Pa, respectively. Similarly, for the PV, these values were noted as 3.52 Pa·s, 16.48 Pa·s, and 4.06 Pa·s, respectively. However, by comparing these values with the NN’s model, they were found to be higher, which also gives confirmation of R-F’s high precision in terms of predicting the outcomes. A validation approach known as k-fold cross validation was also introduced to authenticate the precision of employed models. Moreover, the influence of the input parameters was also investigated with regard to predictions of PV and YS. The proposed study will be beneficial for the researchers and construction industries in terms of saving time, effort, and cost of a project.

## 1. Introduction

Conventional concrete is one of the most widely used building materials in the world [1,2,3]. It is considered a complex substance, which is a mixture of fine and coarse aggregates coupled with a cementitious matrix that is suspended in the air [4,5,6]. However, fresh concrete without enough flowability makes casting, pumping, spreading, and molding around steel reinforcement extremely difficult [7,8,9]. The product’s low strength and poor durability result from inadequate compaction [10,11,12,13,14]. Inadequate cohesion and surface finishing difficulties might also contribute to problems. Fresh concrete must be able to be carried, placed, poured into molds and around reinforcement, compacted, and finished without separating [12,15,16,17,18,19]. To make fresh concrete more fluid, just adding more water is ineffective since the water forms pores that weaken the hardened product [20,21]. Therefore, water-reducing admixtures are desirable and frequently utilized because they can maintain flow at a lower water content, resulting in a substantial boost in concrete’s strength and durability [22,23,24]. For high-rise construction, considerable attention is paid to making concrete pumpable without segregation and bleeding [25,26]. To avoid cold joints, segregation, honeycombing during pumping, transportation, and placement, and compactions around the reinforcement, freshly mixed concrete must have acceptable flowability [27].

Plastic viscosity (PV) refers to the resistance a fluid presents to free flow [28]. This resistance is caused by friction between the deforming liquid and the particles and liquids in the drilling mud [29]. PV is a Bingham plastic model parameter that represents the slope of the shear stress/shear rate line above the yield point [30]. PV is a crucial rheological property that influences the parameters of drilling fluid [31]. However, experimentally and computationally, the yield stress is associated with common field-friendly measuring methods, such as the slump and slump flow test, for evaluating flowability [32].

Rheology is the study of the deformation and flowability of concrete [33,34,35]. It entails measuring yield stress and plastic viscosity at various shear rates and times [36,37,38]. Controlling fresh characteristics enables the production of concrete with the requisite green strength and viscosity [39]. Therefore, the evaluation of fresh characteristics in terms of rheology provides an effective tool for monitoring the requisite flowability in concrete 3-D printing [40,41,42,43]. Even though there have been papers written about constitutive equations that describe how fresh concrete behaves rheologically, only the Bingham model and the Herschel and Bulkley (H–B) model have been accepted [44]. For normal slump concrete, Bingham’s material model has been demonstrated to suit experimental data fairly well and is defined as follows.

(1) $$\;\tau =\tau_{0\;}+\mu_{p}\gamma ˙$$

where τ, $\mu_{p}$, and γ˙ indicates the stress (shear), PV, and shear rate, respectively. Modeling and describing the flow of fresh concrete is based on the assumption that it follows the Bingham model. The rheological properties of concrete have been measured using the same assumption, but the data points are different depending on the testing device [45]. Until a consistent test method for assessing the rheological characteristics of concrete is created, the characterization of concrete’s fresh qualities will remain in a state of uncertainty.

The application of predictive modelling techniques to anticipate the various properties of the objects based on the input parameters is gaining popularity [46,47,48,49,50,51,52], especially supervised predictive machine learning (PML) algorithms, which can predict the required outcome more precisely. Asri et al. [53] also employed various PML techniques to predict the YS and PV of self-compacting concrete (SCC). They used the number of PML approaches on the data set and predicted the aforementioned result. The Ghanbari et al. [54] study was based on the anticipation of PV of self-compacting fiber reinforced concrete. The study reported better precision for the required outcome. AICHA et al. [55] incorporated the NN model and multi-variable regression to anticipate the rheological parameters of the SCC. A total of 59 data points were retrieved from the literature for running the selected models. Yousef et al. [56] employed the ANN model from PML algorithms to predict the YS and PV of the SCC. The experimental data set was developed for running the model for the predictive result. Mohammed et al. [57] study was based on the application of nonlinear regression (NLR) model and ANN model to investigate the predictive outcome for both the rheological properties and strength of cement paste. They reported that the NLR precision level was better as compared to the ANN model toward the prediction

The purpose of the present study is to introduce the soft computing techniques which can successfully predict the complex, time-consuming, and experimental effort-related properties of concrete in a limited time. These novel approaches can help to execute the project work fluently without waiting for the testing results from the laboratory. This research describes the predictive modeling applications of the rheological properties of super-plasticized concrete. In order to avoid bias and increase the robustness of the study, the artificial neural network (NN) machine learning (ML) approach has been selected from the individual ML approaches, while the random forest (R-F) technique has been selected from the ensemble ML approaches for predicting the outcomes. Ensemble ML algorithms normally use weak learners and split the model into 20-sub models for high accuracy. The plastic viscosity (PV) and yield stress (YS) have been investigated with NN and R-F approaches from predictive machine learning (PML). Statistical checks and validation approaches were also adopted to confirm the employed model’s legitimacy. This research is novel in that it describes the effect of both ensemble (R-F) and individual (ANN) PML methods to anticipate the rheological properties of fresh concrete. This research will be beneficial for construction industries by saving time, experimental effort, and money.

## 2. Materials and Data Description

**Figure 1:**
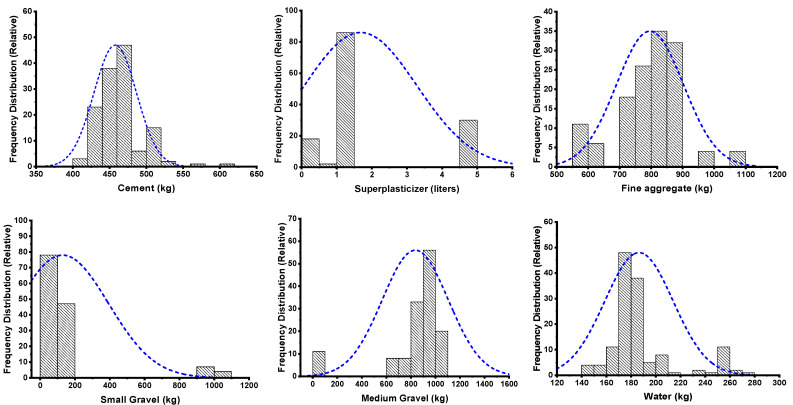
Graphical representation of relative frequency distribution for input parameters.

**Figure 2:**
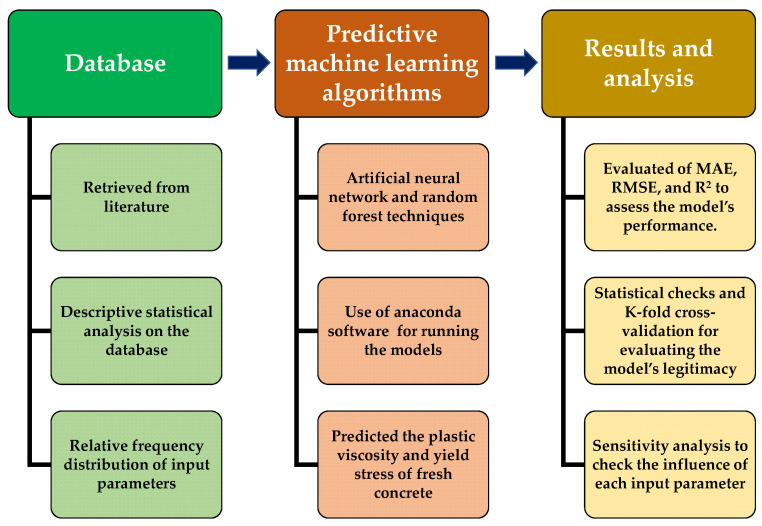
Schematic representation of research’s adopted methodology.

The application of Python coding played a vital role in the required models. The Spyder (4.1.4) of anaconda navigator software was used to introduce the relevant coding of Python for running the employed model [58]. The software adopted the six input parameters, including the sand, cement, water, small gravel, medium gravel, and superplasticizers, while each model ran two times for two different outcomes (PV and YS). The set of data, consisting of 139 data points, used for modelling was retrieved from the literature. Moreover, the coding was set in the software which automatically split the data set into training (80%), and testing (20%) purposes. The relation between the experimental result for both outputs and the predictive result from the modelling were compared. The statistical checks in the form of evaluating the various errors using same software were also applied along with the k-fold cross validation approach to satisfy the accuracy level of the models. In contrast, an additional analysis was carried out (sensitivity analysis) to figure out how much each input parameter affects the prediction of the rheological properties of fresh concrete. The descriptive statistical information of the input variables is listed in tabulated form as shown in Table 1. The histogram in Figure 1 gives the information on the relative frequency distribution of each variable used for running the models. However, the detailed adopted research methodology in the form of the flow chart is presented in Figure 2.

### 2.1. Predictive Modelling Approaches

#### 2.1.1. Artificial Neural Network (NN)

Neural networks (NN) are generally simple and small in size; yet, they feature strong knowledge-and-information-processing capabilities because of their similarities to the human brain [59]. In civil engineering, NNs have been used to find internal damage, identify structural systems, model the behavior of materials, optimize and control structures, monitor underground water, predict how much a shallow foundation will sink, and figure out how much of each ingredient to put in a concrete mix. Input neurons provide the raw content. Weights and biases create connections among input and hidden neurons. Output neurons provide the indication through connections among the hidden and output neurons. Neural networks are used a lot in engineering because they can recognize patterns, learn on their own, organize themselves, and work in real-time. In contrast to many other soft computing techniques, NNs instantly learn from the specified training patterns and builds the relationship between input and output parameters. In addition, NNs impose no constraints on the input parameters for distributions without defined relationships.

#### 2.1.2. Random Forest (R-F)

Leo Breiman proposed R-Fs12 in 2001 as an intelligent combination of classification algorithms based on statistical learning theory [60]. In R-F, the original data are resampled to obtain additional samples mostly via the bootstrap method. After constructing classification trees for each bootstrap sample, the final results are determined by voting on the combined predictions of the classification trees (Figure 1). R-F can be used for both classification and regression applications. It is employed as a regression tool in the current investigation. When utilizing R-F to solve regression problems, the output variables are fitted with values of the input parameters. For each input factor, the data set is divided into many points, and the Sum of Square Error (SSE) is computed at each point for the actual and projected values. The minimum SSE value for this node is then determined. It can also be investigated how important a variable is by switching around all the values of the input variables and measuring how much their accuracy rate changes in the out-of-bag samples (a number of observations that are not used in training and are referred to as the “out-of-bag” data set: OOB data set) [61].

## 3. K-Fold Cross Validation (C-V)

The estimation of prediction accuracy is crucial if our objective is to predict. The training error is a straightforward approximation of the prediction error; however, it is biased downwards. C-V, on the other hand, has an upward bias. The upward bias may be minor in leave-one-out cross validation, but it cannot always be ignored in the computationally preferred 5-fold or 10-fold cross validation. Since the training error has a downward bias and C-V has an upward bias, a family that connects the two estimates will contain an appropriate estimate. Generally, the performance of classification algorithms is tested using C-V. First, a data set is randomly partitioned into k distinct folds with roughly the same number of instances. Then, each fold assumes responsibility for evaluating the model suggested by the other k-1 folds. Throughout this procedure, the training data set is partitioned into multiple ‘k’ smaller pieces. Consequently, the term ‘k’-fold was coined. On the basis of a random data set, k-fold is utilized for testing and k-1 for training. The prediction model’s efficacy is evaluated using a stratified 10-fold cross-validation method. This approach divides the data set into ten folds at random. Consequently, each fold is utilized just once as a validation set. Finally, the error or accuracy measure for each fold can be compared; if they are comparable, the model is likely to generalize well.

## 4. Results and Discussion

### 4.1. Yield Stress Output from NN’s Model

**Figure 3:**
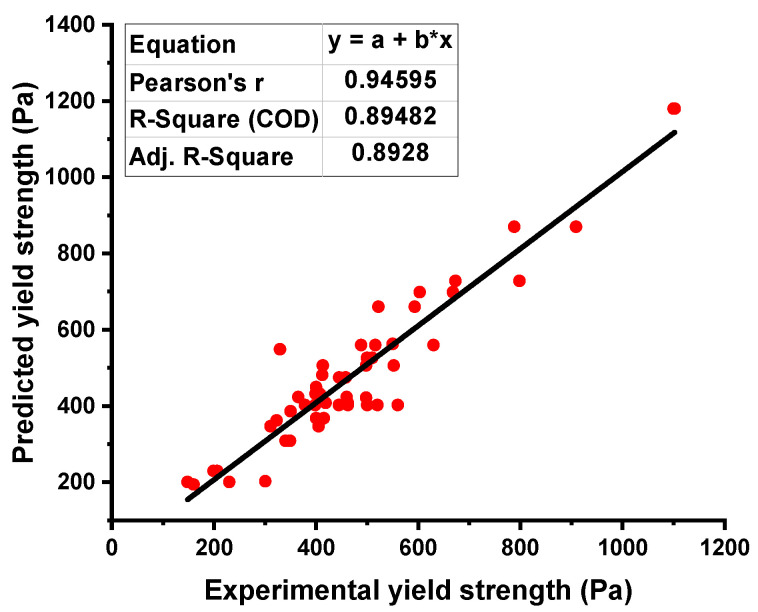
Yield stress relationship for the actual and predicted result of ANN model.

**Figure 4:**
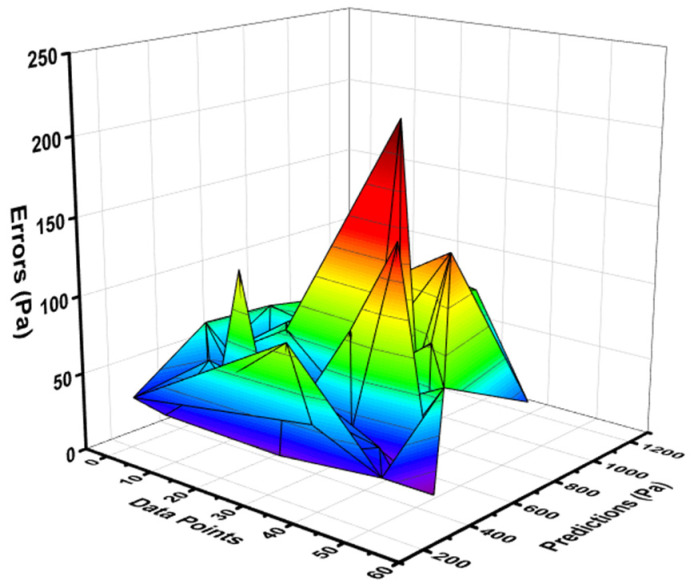
Error distribution of NN model for yield stress.

The relation between the experimental result and the result obtained from the ANN model for YS showed better accuracy, as indicated by the R^2^ value of 0.89, which can be seen in Figure 3. The distribution of the variation between the actual result and ANN’s model output can be seen in Figure 4. This difference gave the maximum and minimum values as 59.85 Pa and 1.35 Pa, respectively. However, it was noted that 25.95% of the variation’s data lied between the minimum value and 30 Pa, and 27.77% of the data were reported between 30 Pa and 50 Pa. However, 42.59% of these data were reported above 50 Pa.

### 4.2. Yield Stress Output from R-F Model

**Figure 5:**
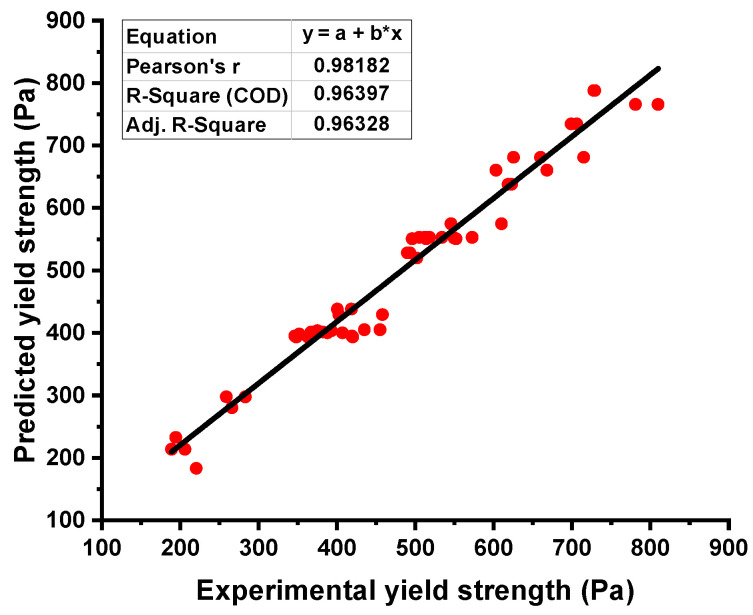
Yield stress relationship for the actual and predicted result of R-F model.

**Figure 6:**
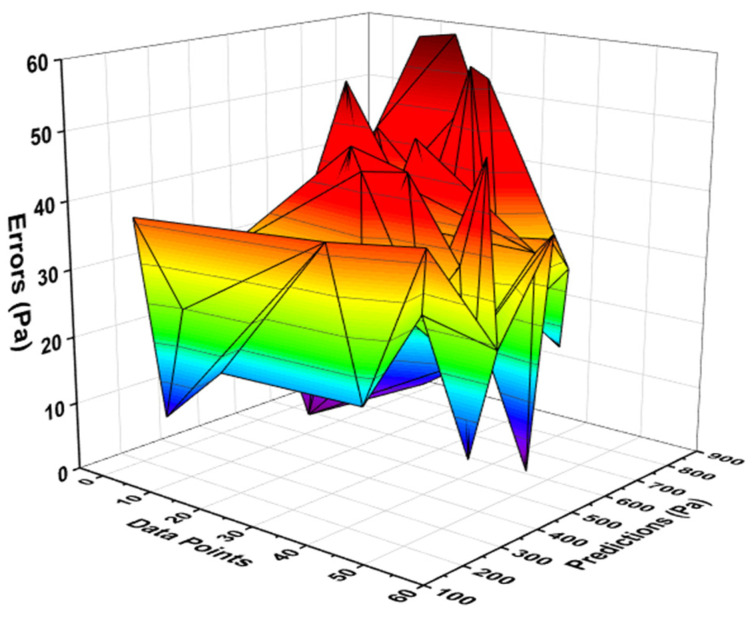
Error distribution of R-F model for yield stress.

R-F model showed strong relation when a comparison was made for the result of YS with the experimental result. R-F gives the R^2^ value equal to 0.96, indicating a much high precision level in terms of predicting the YS of the fresh concrete as opposed to the NN model, as shown in Figure 5. The result of the data representing the difference between the real and forecasted values can be seen in Figure 6. This data showed the maximum, minimum, and average values to be 59.85 Pa, 1.35 Pa, and 30.36 Pa, respectively. It was also noted that 50% of these data were lying between a minimum value of 30 Pa, and 40.74% of the data were reported between 30 Pa to 50 Pa. However, only 9.25% of the aforementioned data were noted above 50 Pa.

### 4.3. Plastic Viscosity Outcome from NN’s Model

**Figure 7:**
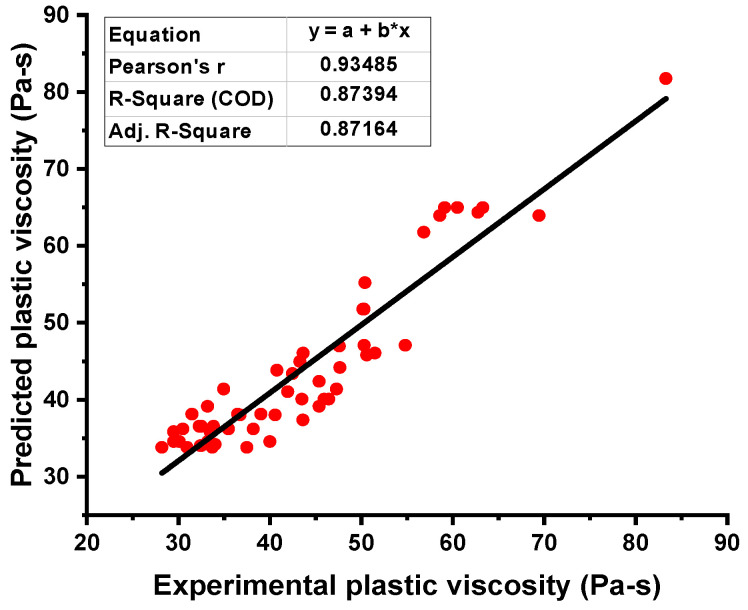
Plastic viscosity relationship for the actual and predicted result of the NN model.

**Figure 8:**
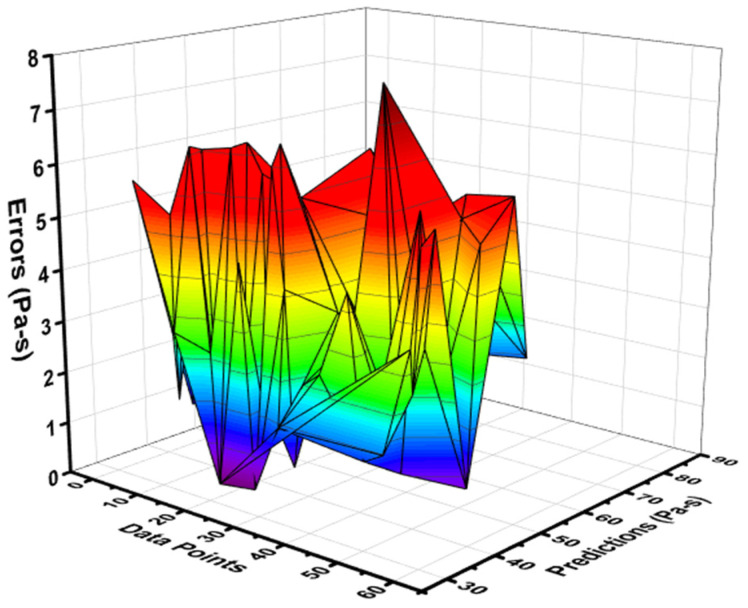
Error distribution of NN model for plastic viscosity.

When comparing the ANN’s model output for the PV of fresh concrete with the experimental result, the precision level in predicting the required result was better. This is indicated by the R^2^ value equal to 0.87, as shown in Figure 7. However, the distribution of the difference values between the experimental and predicted ANN models is depicted in Figure 8. This distribution gives the highest, minimum, and average values as 7.72 Pa·s, 0.11 Pa·s, and 3.52 Pa·s. Moreover, it was noted that 33.33% of these data lie between its minimum value and 2 Pa·s, 35.08% of the data lie between 2 Pa·s and 5 Pa·s, and 31.5% of the difference data were reported above 5 Pa·s.

### 4.4. Plastic Viscosity Outcome from R-F Model

**Figure 9:**
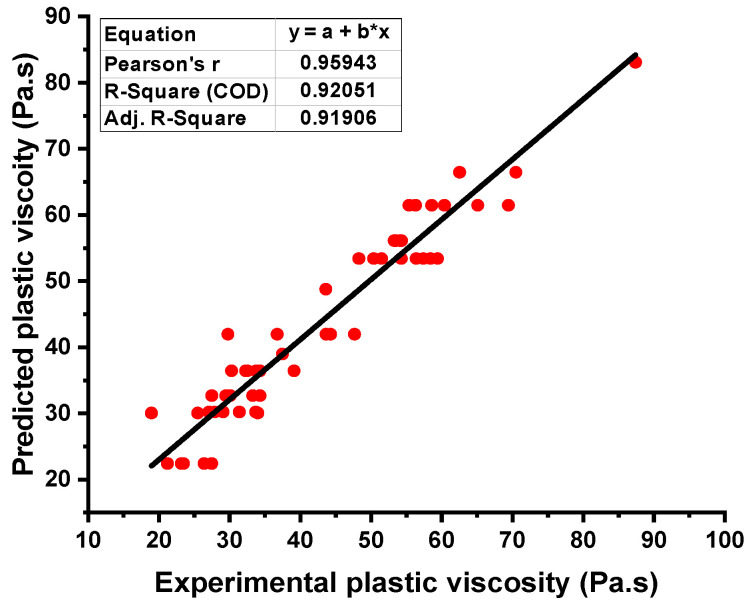
Plastic viscosity relationship for the actual and predicted result of R-F model.

**Figure 10:**
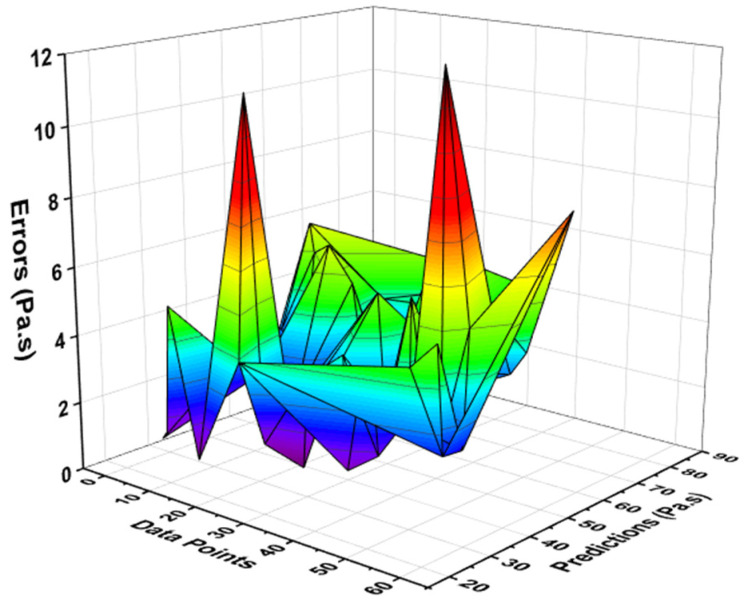
Error distribution of R-F model for plastic viscosity.

The relationship for the PV of fresh concrete between the actual and forecasted results of the R-F model showed high accuracy as opposed to the ANN model. This confirmation was made by examining the coefficient of determination (R^2^) value equal to 0.96 for the R-F model, the reflection of which can be seen in Figure 9. However, the error distribution for the result of PV of fresh concrete between the experimental and predicted outcome is shown in Figure 10. The distribution gives the highest, minimum, and average values equal to 12.18 Pa·s, 0.589 Pa·s, and 3.59 Pa·s, respectively. In addition, 22.80% of these data were reported between the minimum value (0.589 Pa·s) and 2 Pa·s, 52.63% of the data were between 2 Pa·s and 5 Pa·s, while 24.56% of these data were noted above the 5 Pa·s.

## 5. Result of K-Fold Cross Validation (C-V)

C-V is a statistical method for judging or speculating how well machine learning models really work. Because it is important to know how well the chosen models work, users need a validation method to figure out how accurate the model’s data are. For the k-fold validation test, the data set needs to be mixed up randomly and separated into k classes. In this study, experimental sample data were split into 10 subsets. It utilized nine of the ten subgroups, but only one of them was used to test the model. The same part of this process was then performed 10 times to get an average of how accurate these 10 times were. It was clear that the 10-fold cross-validation method gave a good picture of the model’s performance and accuracy.

**Figure 11:**
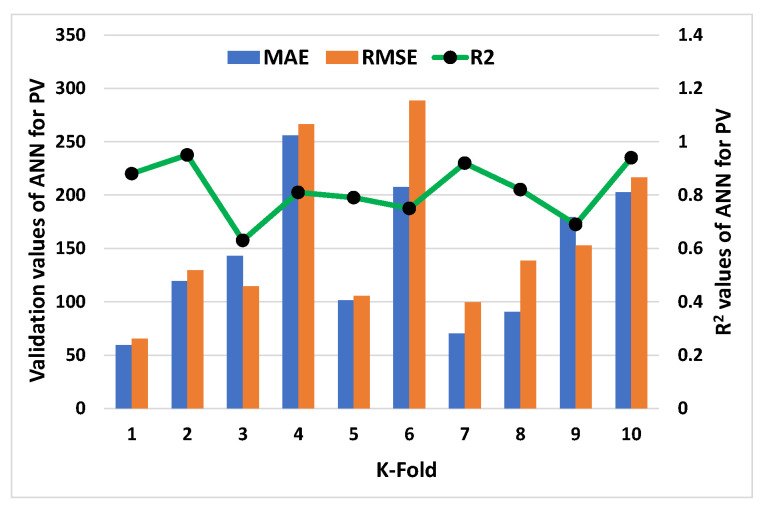
K-fold result of plastic viscosity from ANN model.

**Figure 12:**
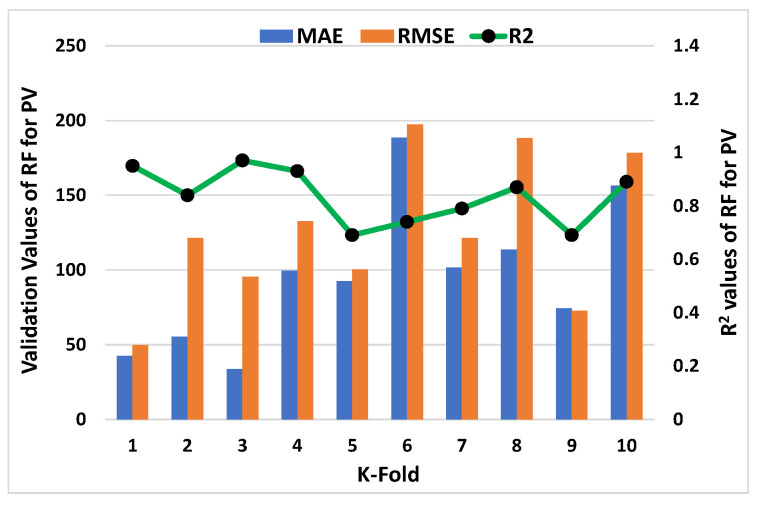
K-fold result of plastic viscosity from R-F model.

**Figure 13:**
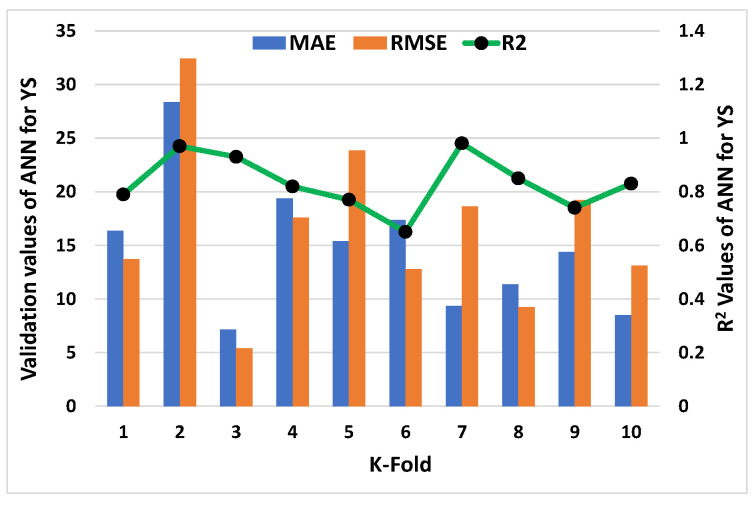
K-fold result of yield stress from NN model.

**Figure 14:**
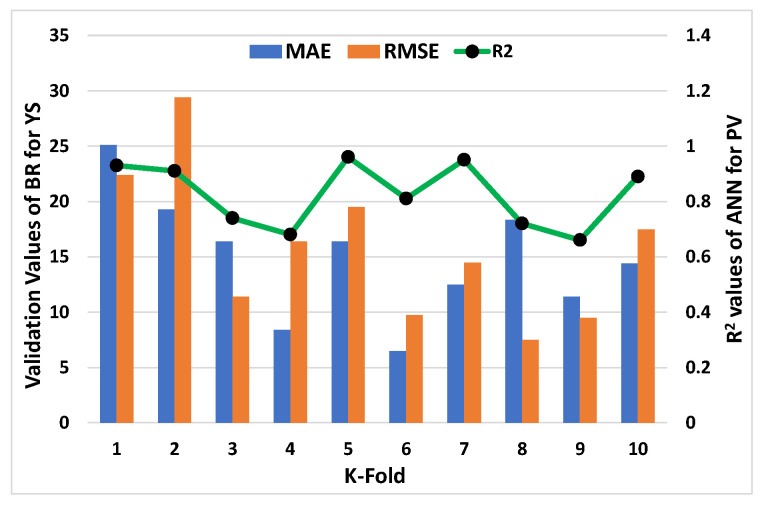
K-fold result of yield stress from R-F model.

C-V could be used to confirm bias and decrease deviation for the data set. Figure 11, Figure 12, Figure 13 and Figure 14 show how a correlation coefficient (R^2^), a mean absolute error (MAE), and a root mean square error (RMSE) were used for both plastic viscosity (PV) and yield stress (YS) to quantify the impact of cross validation. The ANN model’s K-fold C-V for PV gave the highest values for MAE, RMSE, and R^2^ as 255.75 Pa·s, 288.47 Pa·s, and 0.95, respectively, as depicted in Figure 11. A steady increase in MAE in the graph was reported until the k-fold value of 4, while an abrupt decrease was reported at values 5 and 7. Similarly, in the case of R^2^, after a second k-fold value, the minimum result was reported, which seemed to normally increase until the 10th k-fold value. The maximum values of the same parameters for the R-F model to analyze plastic viscosity are 188.48 Pa·s, 147.38 Pa·s, and 0.97, respectively, as shown in Figure 12. In this case, MAE showed a fluctuation in the result until the end point of the graph, while a decrease in the R^2^ result was noted with some variations. Similarly, the maximum values of the ANN’s model for MAE, RMSE, and R^2^ of YS were noted as 28.36 Pa, 32.42 Pa, and 0.98, respectively, as shown in Figure 13. An abrupt increase in MAE was reported at the initial phase, but it showed a sharp decrease at the stage of the 3rd k-fold. Moreover, R^2^ showed a steady decrease until the 6th k-fold and a dramatic increase was reported to reach the maximum value. However, the maximum values for YS of the R-F’s model for the same parameters give 25.1 Pa, 29.39 Pa, and 0.96, respectively, as shown in Figure 14. A steady decrease in MAE was noted in this case until the 4th k-fold value and then fluctuated in the MAE result until the last k-fold. However, the R^2^ result also showed a declining curve until the 4th k-fold value, and then random changes were noted. In contrast, the statistical checks for the YS and PV of concrete for both the models are listed in Table 2 and Table 3, respectively.

## 6. Sensitivity Analysis (S-A) Outcome

**Figure 15:**
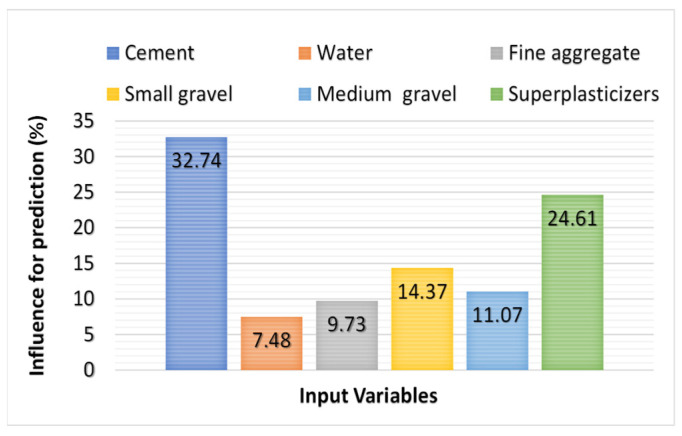
Influence of input parameters on the targeted outcome.

The S-A was introduced to examine the influence and the impact of each input parameter used to determine the predictive outcome for both plastic viscosity (PV) and yield stress (YS). This analysis revealed that the stronger influence on the prediction of rheological parameters was cement, which showed a 32.74% contribution, and superplasticizers with 24.61%. However, other variables contributed less towards the anticipation of rheological parameters of fresh concrete. Contributions made by other variables in descending order were small gravel (14.37%), medium gravel (11.07%), fine aggregate (9.73), and water (7.48%), as shown in Figure 15.

## 7. Discussion

This study examines the use of machine learning techniques to predict the rheological characteristics of fresh concrete. The selection of ANN and R-F models was based on their classification from different types of ML techniques. ANN belongs to the individual ML approach category, while R-F refers to the ensemble ML algorithm. ANN model uses the connection system of neurons and executes the process accordingly for the required output. However, in addition to its basic execution process, twenty R-F sub-models were trained on data and optimized to get the highest R^2^ value. Moreover, the data were also validated by means of K-fold C-V using R^2^, MAE, and RMSE. The input parameters played a vital role in the accuracy level of the employed model. The variation in the result may occur from both increasing or decreasing the total number of input parameters. However, the confirmation, such as statistical checks, sensitivity analysis, and validation for the models, was validated to achieve the precision level.

## 8. Conclusions

This paper proposed a comparison of predictive machine learning (PML) models for the rheological parameters of fresh concrete. The plastic viscosity (PV) and yield stress (YS) properties of the concrete at the initial stage were predicted using artificial neural network (ANN) and random forest (R-F) models. The following conclusions can be drawn from the study:

- The ML algorithms can be successfully employed to anticipate the rheological properties of fresh concrete.
- R-F approach was efficient in predicting both PV and YS of the fresh concrete.
- The proposed model achieved high predictive precision as indicated by the higher coefficient of determination (R^2^) value, equal to 0.92 for PV and 0.96 for the YS of the fresh concrete.
- High predictive accuracy for the R-F model was also confirmed from the statistical checks. The lower values of MAE and RMSE and the high value of R^2^ provided the aforementioned confirmation.
- The input parameter with the highest influence was noted as cement, which contributed 32.74% towards the prediction of rheological parameters of concrete.

The data set can be enhanced with the experimental approach to check the performance level of the models with a large data set. The input parameters can be increased with the addition of the chemicals in concrete, the effect of temperature, the water to cement ratio, and the cement to aggregate ratio. Other ML approaches, such as SVM, Adaboost, XGboost, and deep learning methods, can also be introduced to investigate these properties.

## Figures and Tables

**Table 1:** Statistical description of the concrete’s parameters data set.

Input Variables	Maximum	Minimum	Mean	Standard Deviation
Cement (kg)	604.00	410.00	457.79	28.33
Water (kg)	275.00	147.00	186.32	26.97
Fine aggregate (kg)	1064.00	553.00	796.11	105.09
Coarse gravel (5 × 10 mm)	1010.00	0.00	123.59	258.77
Medium coarse gravel (10 × 20 mm)	1080.00	0.00	840.87	264.80
Superplasticizer (L/100 kg cement)	4.60	0.00	1.80	1.67

**Table 2:** Statistical outcomes of yield stress for employed models.

PML Approaches	MAE (Pa)	MSE (Pa)	RMSE (Pa)
NN Algorithm	54.34	4491.6804	67.02
R-F algorithm	30.36	1141.7641	33.79

**Table 3:** Statistical outcomes of plastic viscosity for employed models.

PML Approaches	MAE (Pa·s)	MSE (Pa·s)	RMSE (Pa·s)
NN Algorithm	3.59	17.9776	4.24
R-F algorithm	3.52	16.4836	4.06

## Data Availability

The data used in this research have been properly cited and reported in the main text.
